# Supplementary figures and images for: DEG-by-Index Ratio Transformation Normalization with Blood RNA-Seq Enhances Early and Consistent Detection of Mouse Tumorigenesis
Source: Biology (Basel). 2025 Nov 11;14(11):1577. doi: 10.3390/biology14111577 (PMC12650743; doi:10.3390/biology14111577)

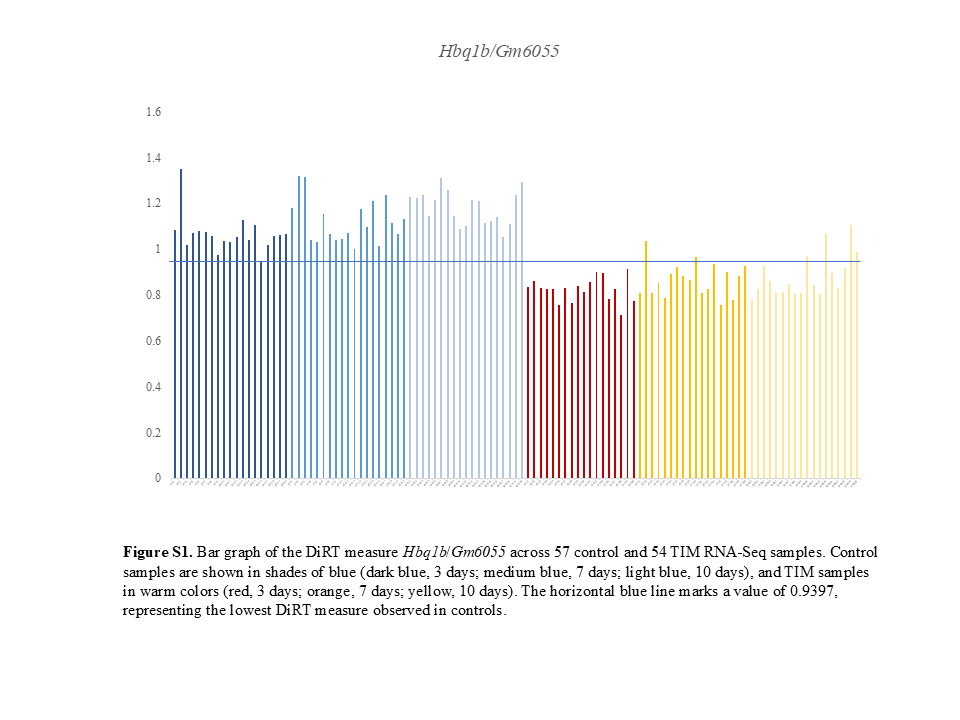

Supplement: Supplementary file 1 [file biology-14-01577-s001.zip › figure S1.tif]

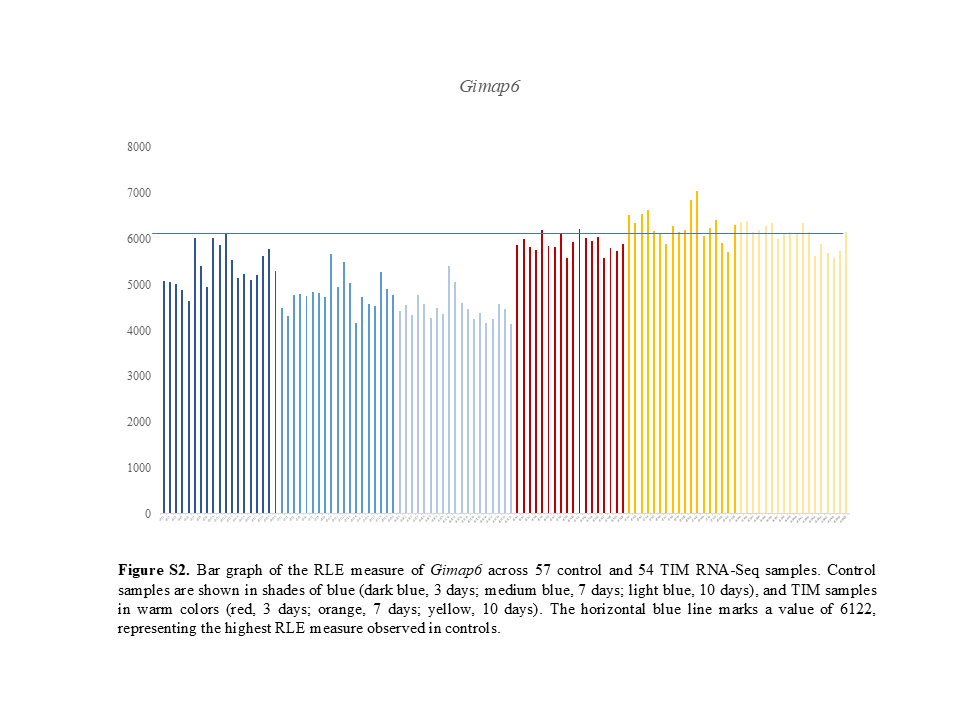

Supplement: Supplementary file 1 [file biology-14-01577-s001.zip › figure S2.tif]
